# Scaled Approach to Designing the Minimum Hybrid Reinforcement of Concrete Beams

**DOI:** 10.3390/ma13225166

**Published:** 2020-11-16

**Authors:** Andrea Gorino, Alessandro P. Fantilli

**Affiliations:** 1 Rete Ferroviaria Italiana S.p.A., Via Nizza 2, 10125 Torino, Italy; a.gorino@rfi.it; 2 Department of Structural Geotechnical and Building Engineering, Politecnico di Torino-DISEG, Corso Duca degli Abruzzi 24, 10129 Torino, Italy

**Keywords:** reinforced concrete (RC), fiber-reinforced concrete (FRC), hybrid-reinforced concrete (HRC), rebar, fibers, bending moment, minimum reinforcement, ductility index

## Abstract

To study the brittle/ductile behavior of concrete beams reinforced with low amounts of rebar and fibers, a new multi-scale model is presented. It is used to predict the flexural response of an ideal Hybrid Reinforced Concrete (HRC) beam in bending, and it is validated with the results of a specific experimental campaign, and some tests available in the technical literature. Both the numerical and the experimental measurements define a linear relationship between the amount of reinforcement and the Ductility Index (DI). The latter is a non-dimensional function depending on the difference between the ultimate load and the effective cracking load of a concrete beam. As a result, a new design-by-testing procedure can be established to determine the minimum reinforcement of HRC elements. It corresponds to DI = 0, and can be considered as a linear combination of the minimum area of rebar (of the same reinforced concrete beam) and the minimum fiber volume fraction (of the same fiber-reinforced concrete beam), respectively.

## 1. Introduction

In the technical literature, there is a strong interest in the use of Hybrid Reinforced Concrete (HRC) structures, reinforced by both steel bars and discrete fibers randomly dispersed in the cementitious matrix [1,2,3,4,5]. In addition to rigid pavements [6], HRC is often adopted in massive concrete members (e.g., precast and cast-in-situ tunnel linings) with the aim of reducing the amount of traditional rebar [7,8,9,10,11]. This is possible in structural elements containing low amounts of reinforcement, because the increment in strength provided by the fibers is comparable to that of rebar [12].

**Figure 1:**
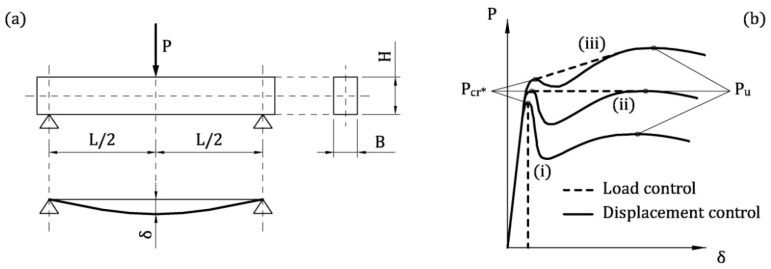
Flexural behavior of concrete beam reinforced with rebar and/or fibers: (**a**) three point bending test; (**b**) applied load vs. midspan deflection curves in the cases of (i) brittle response, (ii) brittle/ductile transition, and (iii) ductile response.

In concrete beams in bending (Figure 1a), having a massive cross-sectional area, the curve load *P* vs. midspan deflection δ (Figure 1b) can show an ultimate load *P*_u_ smaller than the effective cracking load *P*_cr_* [8,9]. To be more precise, *P*_u_ corresponds to the failure of the reinforcing system (made of rebar and/or fibers), whereas *P*_cr_* is the load that produces the first crack [13]. In the post-cracking stage of load controlling tests, if the reinforcement cannot bear the load carried by concrete, brittle failure occurs, as showed by the dashed curve (i) in Figure 1b. On the contrary, ductile failure of lightly reinforced concrete beams is guaranteed (see the curves (ii), (iii) of Figure 1b) when

(1) $$P_{u}\geq P_{\mathrm{cr}*}$$

In Lightly Reinforced Concrete (LRC) beams, the brittle failure is avoided by introducing a minimum reinforcement *A*_s,min_, defined as the amount of steel rebar which guarantees the condition *P*_u_ = *P*_cr_* [14,15,16,17]. The minimum reinforcement of LRC members should also ensure crack control in service [18], hence *A*_s,min_ provided by building codes generally fulfill both the ultimate (static) and the serviceability limit states [18,19,20]. Similarly, in Fiber-Reinforced Concrete (FRC) beams, the minimum fiber volume fraction *V*_f,min_ can be defined as *A*_s,min_ in LRC beams [21,22,23]. In other words, when the content of fiber is *V*_f,min_, the transition from deflection-softening (i.e., the brittle response) to deflection-hardening (i.e., the ductile response) occurs [23,24].

If the minimum reinforcement of HRC beams is evaluated as in those LRC are, the resisting contribution of fibers is not exploited, making the use of fiber-reinforcement useless. This is particularly true in the case of massive structures, when the computation of *A*_s,min_, performed in accordance with building code rules, leads to a large amount of steel rebar [8].

Even in several projects focused on the flexural behavior of HRC members, little research has been devoted, to date, to studying the problem of minimum hybrid reinforcement. For instance, the experimental campaigns performed by Barros et al. [25], Blanco et al. [26], Meda et al. [27], and Ning et al. [28] involved only beams with deflection hardening. On the other hand, Carpinteri et al. [29], Dancygier and Berkover [30], di Prisco et al. [31], Dupont [32], Falkner and Henke [33] and You et al. [34] analyzed under-reinforced concrete beams, without focusing on the minimum hybrid reinforcement.

The transition from the brittle to ductile behavior of HRC members was investigated deeply by Chiaia et al. [35], Liao et al. [3] and Mobasher et al. [36]. Although they demonstrated the possibility of reducing the amount of steel rebar in presence of fibers, a simple and univocal criterion for evaluating the minimum reinforcement of HRC beams cannot be found in the current literature. Thus, the introduction of a criterion for evaluating the minimum amount of hybrid reinforcement, made with rebar and fibers, is of practical interest. To fill this research gap, both numerical and experimental investigations on lightly reinforced HRC beams are performed herein. In particular, an approach to evaluate the minimum reinforcement ratio of HRC beams is described in the following sections. It can be considered as an extension to HRC of the design-by-testing procedure already proposed by Fantilli et al. [15,21,37] for LRC and FRC beams. The effectiveness of this new approach is corroborated not only by the experimental data measured by other authors, but also by new tests performed on 30 concrete beams reinforced with low amounts of rebar and/or fibers.

## 2. Multi-Scale Model for HRC Beams

**Figure 2:**
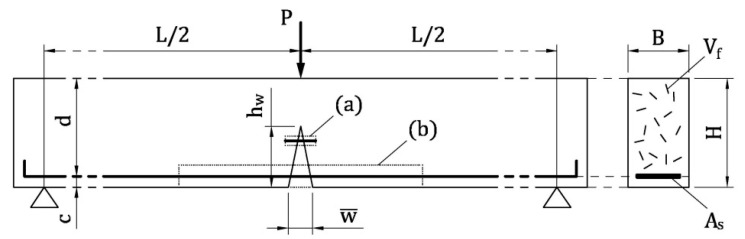
Modelling the bridging effect of the reinforcing systems in a Hybrid Reinforced Concrete (HRC) beam under bending actions: (**a**) the ideal tie representing the fiber and the surrounding cementitious matrix; (**b**) the ideal tie representing the rebar surrounded by Fiber-Reinforced Concrete (FRC).

**Figure 3:**
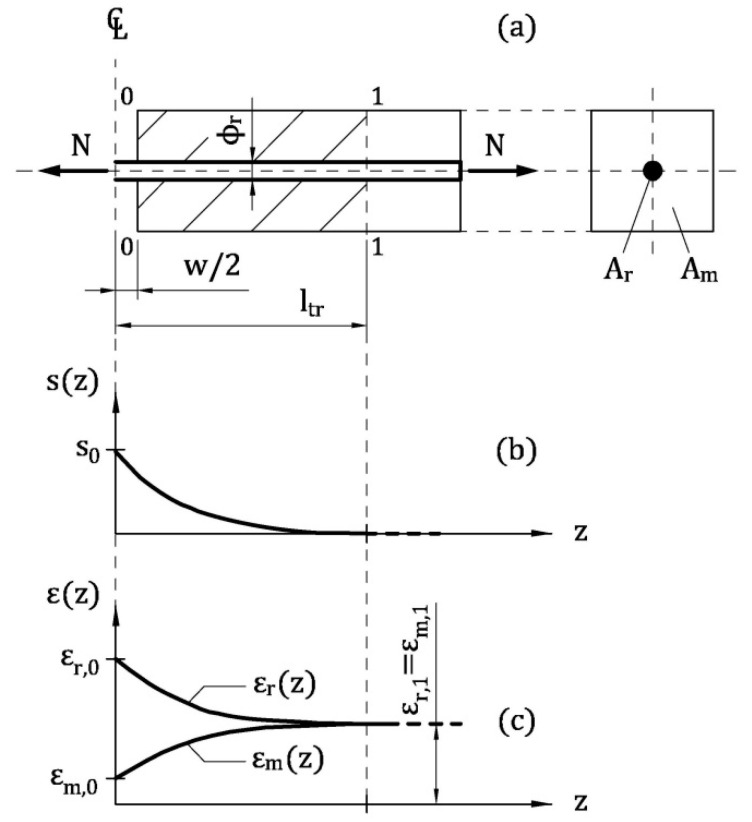
The ideal tie composed by the reinforcement and the surrounding cement-based material: (**a**) longitudinal and transversal cross-section; (**b**) slip between reinforcement and matrix; (**c**) strains in reinforcement and concrete.

A new general model is introduced herein to predict the flexural behavior of HRC beams. Specifically, both the bridging effects on crack surfaces given by rebar and fibers are analyzed through ideal ties (Figure 2), composed by the reinforcement and the surrounding cementitious matrix. This tie has a single orthogonal crack in the midsection (Figure 3a). At the micro scale (Figure 2a), the pull-out mechanism of the fiber (i.e., the solution of the tension-stiffening problem) provides the cohesive stress vs. crack width relationship of FRC. This relationship represents the response of cracked FRC surrounding the rebar of the HRC beam. The tension-stiffening problem can also be solved at the scale of the beam, in the rebar–FRC tie (Figure 2b), obtaining the flexural response of this structural element. Different from other approaches (see, for instance, Barros et al. [38]), in the proposed multi-scale model the strain localization of concrete in compression is neglected, because it generally does not occur in the presence of a low amount of reinforcement.

### 2.1. Formulation of the Tension-Stiffening Problem

To evaluate the post-cracking behavior of the reinforced concrete tie depicted in Figure 3a, a portion of this element, delimited by the cracked cross-section and the so-called Stage I cross-section, is investigated [15]. To be more precise, the cracked cross-section, labelled as 0-0 in Figure 3a, is assumed to be in the midspan of the tie and orthogonal to the reinforcement. Moreover, in the Stage I cross-section (i.e., the cross-section 1-1 in Figure 3a) the perfect bond between reinforcement and cementitious matrix is re-established. Within *l*_tr_ (= transfer length, which the distance between the cross-sections 0-0 and 1-1), as the horizontal coordinate *z* increases, tensile stresses (and strains) transfer from reinforcement to the matrix, due to the bond-slip mechanism acting at the interface of the materials. The slip *s* between reinforcement and matrix vanishes in the Stage I cross-section (Figure 3b), where the strains of reinforcement ε_r_ and of matrix ε_m_ are both equal to that computed with the linear elastic theory (ε_r,1_ = ε_m,1_ in Figure 3c). At the scale of fiber (Figure 2a) and of beam (Figure 2b), the subscripts *r* = *f* = steel fiber and *r* = *s* = steel rebar, respectively. Similarly, *m* = *t* = cement-based matrix surrounding the fiber in tension and *m* = *c* = FRC matrix surrounding the rebar.

When 0 ≤ *z* < *l*_tr,_ the interaction between reinforcement and matrix is described by the following equilibrium and compatibility equations

(2) $$\frac{d\sigma_{r}}{dz}=-\frac{p_{r}}{A_{\;r}}\cdot \tau \left[s\left(z\right)\right]=-\frac{4}{\phi_{\;r}}\cdot \tau \left[s\left(z\right)\right]$$

(3) $$\frac{ds}{dz}=-\left[\varepsilon_{r}\left(z\right)-\varepsilon_{m}\left(z\right)\right]$$

where σ_r_ = stress in the reinforcement; *p*_r,_ *A*_r,_ ϕ_r_ = perimeter, area, and diameter of the reinforcement cross-section;τ = bond stress corresponding to the slip *s* between reinforcement and matrix.

Equations (2) and (3) are the equations of the tension-stiffening problem, which can be solved by introducing the following boundary conditions:

- In the cracked cross-section (at *z* = 0), the slip *s*_0_ = *w*/2, where *w* = crack width at level of reinforcement;
- At z = *l*_tr_ < *L*_r/_2 (where *L*_r_ = reinforcement length), *s* = 0 and d*s*/d*z* = 0 have to be imposed.

Moreover, both at the scale of fiber and at that of the beam, the bond-slip between reinforcement and cementitious matrix and the fracture mechanics of concrete in tension need to be considered [39].

#### 2.1.1. At the Scale of Fiber

According to Fantilli et al. [21], in the ideal tie reproducing FRC (Figure 2a), fiber-reinforcement is a single straight steel fiber surrounded by the cross-sectional area of concrete matrix *A*_t,_ which, is in turn, a function of the fiber volume fraction *V*_f_

(4) $$A_{\;t}=\frac{A_{\;f}}{V_{f}}=\frac{\pi \cdot \phi_{\;f}{}^{2}}{4\cdot V_{f}}$$

where *A*_f,_ ϕ_f_ = area and diameter of the fiber cross-section, respectively.

**Figure 4:**
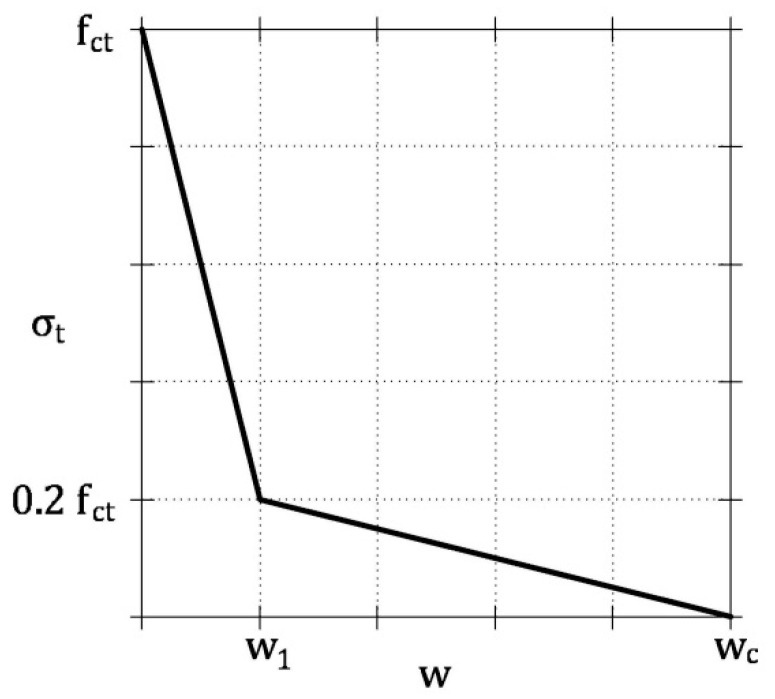
Fictitious crack model proposed by Model Code 2010 [20].

Both steel fiber and uncracked matrix are assumed in the linear elastic regime, whereas the cohesive stresses on the crack surfaces of the ideal tie are defined by the fictitious crack model shown in Figure 4. It consists of a bilinear stress vs. crack width relationship, σ_t_-*w*, as proposed by Model Code 2010 [20]

(5a) $$\sigma_{t}=f_{\mathrm{ct}}\cdot \left(1.0-0.8\cdot \frac{w}{w_{1}}\right)\;for\;0\;<\;w\;\leq \;w_{1}$$

(5b) $$\sigma_{t}=f_{\mathrm{ct}}\cdot \left(0.25-0.05\cdot \frac{w}{w_{1}}\right)\;for\;w_{1}\;<\;w\;\leq \;w_{c}$$

where *w*_1_ = *G*_F/_*f*_ct_; *w*_c_ = 5 ∙ *G*_F_/*f*_ct_; *G*_F_ = 0.073 × *f*_c_^0.18^ = fracture energy of concrete in tension in N/mm (*f*_c_ = compressive strength of concrete in MPa); and *f*_ct_ = tensile strength of concrete (in MPa).

Model Code 2010 [20] also suggests the mean value of *f*_ct_, which can be estimated from the compressive strength (expressed in MPa)

(6a) $$f_{\mathrm{ct}}=0.3\cdot {\left(f_{c}-8\;\right)}^{2/3}\;for\;fc_{\;}\leq \;58\;\mathrm{MPa}$$

(6b) $$f_{\mathrm{ct}}=2.12\cdot \ln\left(\;1+0.1\cdot f_{c}\right)\;for\;fc_{\;}>\;58\;\mathrm{MPa}$$

Moreover, the interaction between fiber and concrete matrix is modelled with a bond-slip τ s relationship. For the sake of the simplicity, the model proposed by Fantilli and Vallini [40], originally developed for smooth steel fibers in a cementitious matrix, is adopted herein

(7a) $$\tau =\tau_{\max}\cdot {\left(\frac{s}{s_{1}}\right)}^{\alpha }\;for\;0\;\leq \;s\;<\;s_{1}$$

(7b) $$\tau =\tau_{f}+\left(\tau_{\max}-\tau_{f}\right)\cdot e^{\beta \cdot \left(s_{1}-s\right)}\;for\;s_{1}\;\leq \;s$$

where τ_max_ = maximum bond stress; τ_f_ = residual bond stress; *s*_1_ = 0.1 mm; α = 0.5; and β = 2/mm.

The values of τ_max_ and τ_f_ can be computed with the following formulae [40]

(8a) $$\tau_{\max}=\frac{1.572}{\sqrt{12.5+\phi_{\;f}}}\cdot \sqrt{f_{c}}$$

(8b) $$\tau_{f}=0.1\cdot \sqrt{f_{c}}$$

where the compressive strength of concrete is in MPa and the diameter of fiber is in mm.

With all these data, the tension-stiffening problem is solved within the domain 0 ≤ *z* < *l*_tr_, by using the iterative procedure summarized in the following points [21]:

1. Assign a value to the crack width *w* in the midsection of the ideal tie (Figure 3a);
2. Compute the slip *s*_0_ in the midsection (at *z* = 0 in Figure 3b)
  (9) $$s_{0}=\frac{w}{2}$$
3. By means of Equation (5), calculate the tensile stress of the matrix in the midsection σ_t,0_;
4. Under the hypothesis of linear elastic behavior of the concrete matrix, calculate the strain in midsection ε_t,0_ (with *E*_t_ = modulus of elasticity of concrete matrix)
  (10) $$\varepsilon_{t,0}=\frac{\sigma_{t,0}}{E_{t}}$$
5. Assume a trial value to the axial load *N* (Figure 3a);
6. By imposing the equilibrium in the cracked cross-section, the stress in the fiber σ_f,0_ can be evaluated with the following equation
  (11) $$\sigma_{f,0}=\frac{N-\sigma_{t,0}\cdot A_{\;t}}{A_{\;f}}$$
7. Assuming the linear elastic behavior of the fiber (σ_f,0_ has to be lower than *f*_u_, where *f*_u_ = strength of fiber), calculate the strain in midsection ε_f,0_
  (12) $$\varepsilon_{f,0}=\frac{\sigma_{f,0}}{E_{f}}$$
  where *E*_f_ = modulus of elasticity of the fiber.
8. Considering Δ*l* as a small portion of the unknown *l*_tr_, define *z*_i_ = *i* ∙ Δ*l* (where i = 1, 2, 3, …);
9. For each *i* (or *z*_i_) calculate:
  - The bond stress τ_i,_ related to the slip *s*_i-1_ [Equation (7)];
  - The strain of the fiber ε_f,i,_ based on Equation (2)
    (13) $$\varepsilon_{f,i}=\varepsilon_{f,i\;-1}-\frac{4}{\phi_{\;f}\cdot E_{f}}\cdot \tau_{i}\cdot \Delta l$$
  - The strain ε_t,i_ of the matrix
    (14) $$\varepsilon_{t,i}=\frac{N-\varepsilon_{f,i}\cdot E_{f}\cdot A_{\;f}}{E_{t}\cdot A_{\;t}}$$
  - The slip *s*_i_ by means of the finite difference form of Equation (3):
    (15) $$s_{i}=s_{i\;-1}-\left(\varepsilon_{f,i}-\varepsilon_{t,i}\right)\cdot \Delta l$$
10. When *s*_i_ ≅ 0, if ε_f,i_ ≠ ε_t,i_ change *N* and go back to step 6;
11. Calculate the tensile stress σ_c_ referred to the cross-sectional area of the tie (i.e., *A*_t_ + *A*_f_)
  (16) $$\sigma_{c}=\frac{N}{A_{\;t}+A_{\;f}}$$

For a given *w*_,_ such a procedure calculates the corresponding stress of the cracked FRC. The complete σ_c_-*w* curve can be obtained by varying the assigned crack width.

#### 2.1.2. At the Scale of Beam

As in the case of LRC members [15], a block of HRC beam in three-point bending, which fails in the presence of a single flexural crack, is modelled. Within such a block, an ideal tie including the steel rebar in tension and the surrounding FRC can be identified (Figure 2b). In analogy with the fiber scale, this tie is delimited by the cracked cross-section (i.e., the midsection 0-0 in Figure 3) and the Stage I cross-section (i.e., the cross-section 1-1 in Figure 3), in which the perfect bond between rebar and FRC in tension is present.

In accordance with Chiaia et al. [35], strain decrements in rebar and strain increments in concrete at level of reinforcement can be assumed as

(17a) $$\varepsilon_{s}\left(z\right)=\varepsilon_{s,0}-\chi \left(z\right)\cdot \left(\varepsilon_{s,0}-\varepsilon_{s,1}\right)$$

(17b) $$\varepsilon_{c}\left(z\right)=\varepsilon_{c,0}-\chi \left(z\right)\cdot \left(\varepsilon_{c,0}-\varepsilon_{c,1}\right)$$

where ε_s_ and ε_c_ = strains in steel rebar and concrete at level of reinforcement; ε_s,0_ and ε_c,0_ = values of ε_s_ and ε_c_ in the cracked cross-section; ε_s,1_ and ε_c,1_ = values of ε_s_ and ε_c_ in the Stage I cross-section (evaluated according to the linear elastic theory); and χ = coefficient of similarity.

**Figure 5:**
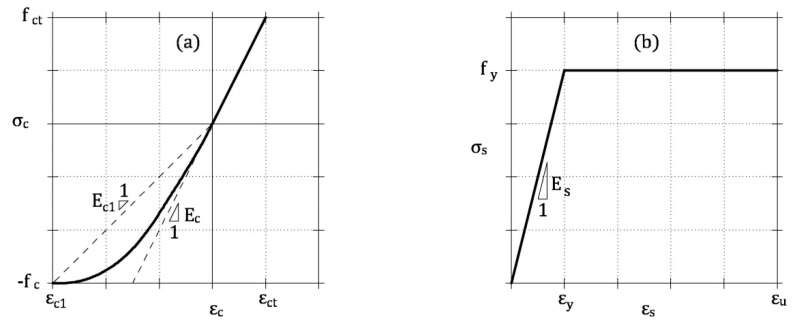
Constitutive relationships for the materials of an HRC beam [20]: (**a**) ascending branch of the Sargin’s parabola for concrete in compression, and linear elastic law for uncracked concrete in tension; (**b**) elastic-perfectly plastic law for steel rebar.

In uncracked concrete, linear elastic constitutive law is assumed in tension, whereas the ascending branch of the Sargin’s parabola [20] is the σ_c-_ε_c_ relationship in compression (Figure 5a)

(18) $$\sigma_{c}=-f_{c}\cdot \left[\frac{k\cdot \eta -\eta^{2}}{1+\left(k-2\right)\cdot \eta }\right]\;for\;\varepsilon_{c1}\;<\;\varepsilon_{c\;}\leq \;0$$

where *k* = *E*_c/_*E*_c1_ = plasticity number; *E*_c_ = 21,500 × (*f*c/10) ^1/3^ = tangent modulus of elasticity of concrete, at the origin of the stress (*f*_c_ in MPa); *E*_c1_ = *f*_c_/ε_c1_ = secant modulus from the origin to the peak in compressive stress; ε_c1_ = strain at the peak in stress; η = ε_c_/ε_c1_ = normalized compressive strain.

The stress vs. strain relationship σ_s-_ε_s_ of the steel rebar is modeled with the elastic-perfectly plastic constitutive law illustrated in Figure 5b [20]

(19a) $$\sigma_{s}=E_{\;s}\cdot \varepsilon_{s}\;for\;0\;\leq \;\varepsilon_{s}\;<\;\varepsilon_{y}\;=\;fy_{/}E_{s}$$

(19b) $$\sigma_{s}=f_{y}\;for\;\varepsilon_{y}\;=\;fy_{/}Es_{\;}\leq \;\varepsilon_{s}\;<\;\varepsilon_{u}$$

where *E*_s,_ *f*_y,_ ε_y_ and ε_u_ are the modulus of elasticity, the yielding strength and strain, and the ultimate strain of steel rebar, respectively.

**Figure 6:**
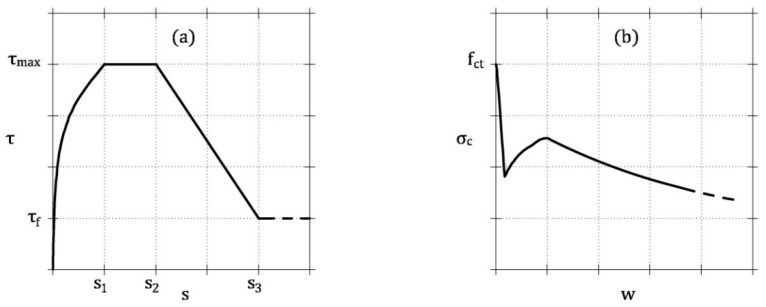
Stresses at the interface between rebar and concrete in tension and on the crack surface: (**a**) bond-slip model proposed by Model Code 2010 [20]; (**b**) stress vs. crack width relationship obtained from the analysis at the scale of the fiber.

To describe the interaction at the interface between rebar and concrete, the bond–slip relationship proposed by Model Code 2010 [20] for ribbed bars is used (see Figure 6a)

(20a) $$\tau =\tau_{\max}\cdot {\left(\frac{s}{s_{1}}\right)}^{\alpha }\;for\;0\;\leq \;s\;<\;s_{1}$$

(20b) $$\tau =\tau_{\max}\;for\;s_{1}\;\leq \;s\;<\;s_{2}$$

(20c) $$\tau =\tau_{\max}-\left(\tau_{\max}-\tau_{f}\right)\cdot \frac{s-s_{2}}{s_{3}-s_{2}}\;for\;s_{2}\;\leq \;s\;<\;s_{3}$$

(20d) $$\tau =\tau_{f}\;for\;s_{3}\;\leq \;s$$

where τ_max_ = 2.5 × *f*_c_^0.5^ (*f*_c_ in MPa); τ_f_ = 0.4 ∙ τ_max_; α = 0.4; *s*_1_ = 1.0 mm; *s*_2_ = 2.0 mm; and *s*_3_ = *c*_clear_ = clear distance between the ribs of rebar.

Finally, the fictitious crack model obtained with the procedure described at fiber scale, and depicted in Figure 6b, is adopted to model the behavior of the cracked FRC.

What follows is the procedure used to solve the tension-stiffening problem at level of rebar [15]:

1. In the cracked cross-section, assign a value to the crack width at the bottom level $\bar{w}$ (Figure 2);
2. Assume a trial value for the crack depth *h*_w_ (*c* < *h*_w_ < *H* in Figure 2, where *c* = concrete cover, and *H* = beam depth);
3. Assuming a linear crack profile (Figure 2), calculate the slip *s*_0_ in the cracked cross-section (where *z*_i_ = 0)
  (21) $$s_{0}=\frac{\bar{w}}{2}\cdot \frac{h_{w}-c}{h_{w}}$$
4. Calculate the cohesive stress σ_c,0_(*w*) in cracked FRC at *z* = 0 by means of the stress vs. crack opening relationship obtained by modelling the ideal tie at the scale of the fiber (Figure 6b);
5. In cross-section 0-0, assume a plane state of strain for uncracked FRC, and calculate ε_c,0_ and ε_s,0_;
6. In the same cross-section, define the state of stress of uncracked FRC, σ_c,0_, and of steel rebar, σ_s,0_, by means of Equations (18) and (19);
7. Calculate the result of axial stresses *R* in the cracked cross-section;
8. If *R* ≠ 0, then change the state of strain and go back to step 6;
9. Compute the internal bending moment *M* in the cracked cross-section;
10. Considering Δ*l* as a small portion of the transfer length, define *z*_i_ = *i* ∙ Δ*l* (where *i* = 1, 2, 3, …);
11. For each *i* (or *z*_i_), calculate:
  - The bond stress τ_i,_ related to the slip *s*_i-1_ (Equation (20));
  - The strain ε_s,i_ in the reinforcement, by using Equation (2) written in the finite difference form (where ϕ_s_ = diameter of rebar)
    (22) $$\varepsilon_{s,i}=\varepsilon_{s,i\;-1}-\frac{4}{\phi_{\;s}\cdot E_{\;s}}\cdot \tau_{i}\cdot \Delta l$$
  - The similarity coefficient χ_i,_ by inverting Equation (17a)
    (23) $$\chi_{i}=\frac{\varepsilon_{s,0}-\varepsilon_{s,i}}{\varepsilon_{s,0}-\varepsilon_{s,I}}$$
  - The strain of concrete ε_c,i_ at level of rebar with Equation (17b);
  - The slip *s*_i_ by means of the finite difference form of Equation (3)
    (24) $$s_{i}=s_{i\;-1}-\left(\varepsilon_{s,i}-\varepsilon_{c,i}\right)\cdot \Delta l$$
12. When *s*_i_ ≅ 0, if ε_s,i_ ≠ ε_c,i_, change *h*_w_ and go back to step 3.

The previous procedure calculates the internal moment *M* corresponding to a given $\bar{w}$ and, consequently, the complete *M*-$\bar{w}$ by varying the assigned crack width.

## 3. Numerical Investigation

In what follows, the M-$\bar{w}$ curves of several ideal HRC beams in bending are numerically computed. The aim is to evaluate the effects of reinforcement (i.e., rebar and/or fibers) on the brittle/ductile behavior of some hybrid beams, in order to identify the condition of minimum reinforcement. More precisely, 108 ideal HRC beams in three-point bending are taken into consideration. They are divided into 36 groups of three beams, having the same geometrical and material properties, but with different amounts of rebar or fibers. In particular, in 18 groups the area of rebar changes and the fiber volume fraction is constant, whereas in the remaining 18 groups *A*_s_ is the same and *V*_f_ varies. For all the groups, the width *B* and the span *L* of the beams are 0.5 and 6 times the depth *H* (which is equal to 200 and 400 mm), respectively. Three compressive strengths of concrete are considered (i.e., *f*_c_ = 30, 45, and 60 MPa), and *E*_t_ = *E*_c_ in all the beams. The same properties of steel rebar are assumed in all the groups (i.e., *f*_y_ = 450 MPa, and *E*_s_ = 210 GPa), whereas steel fibers (with *L*_f_ = 60 mm, *f*_u_ = 1000 MPa, and *E*_f_ = 210 GPa) have the aspect ratio *L*_f/_ϕ_f_ = 40, 60, and 80. Hence, for each group of HRC members, the minimum amount of reinforcement *A*_s,min_, and of *V*_f,min_, defined by Equation (1) in the presence of sole rebar, or sole fibers, are known. They are computed by applying the design-by-testing procedure proposed by Fantilli et al. [15,21,37] for LRC and FRC elements.

Table 1 summarizes the characteristics of all the beams, which are labeled with the acronym SX_CYY_AZZ_ϕW_K, where X depends on the beam depth (X = 1 for H = 200 mm, and X = 2 for H = 400 mm), YY is the concrete strength in MPa, ZZ is the fiber aspect ratio, W is the rebar diameter in mm, and K is a number (1, 2, or 3) associated with the different amounts of hybrid reinforcement in each of the 36 groups.

**Figure 7:**
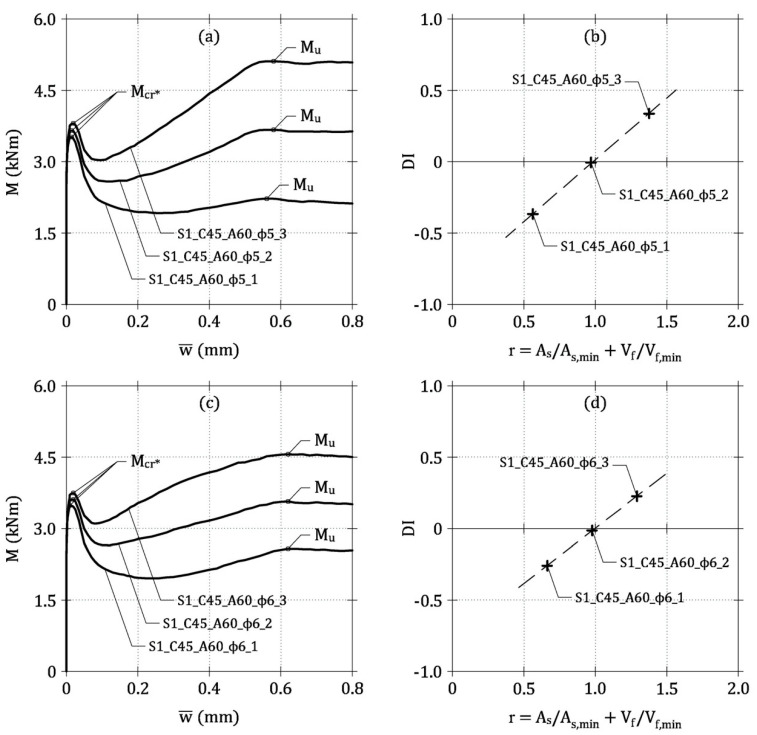
Application of the general model: (**a**) *M*-$\bar{w}$ curves of the ideal beams of Group 9; (**b**) *DI*-*r* relationship of the ideal beams of Group 9; (**c**) *M*-$\bar{w}$ curves of the ideal beams of Group 10; (**d**) *DI*-*r* relationship of the ideal beams of Group 10.

As an example, the *M*-$\bar{w}$ curves of the three beams of Group 9 are reported in Figure 7a. Two stationary points, concerning the effective cracking moment (*M*_cr_*) and the ultimate bending moment (*M*_u_), are clearly evident in each curve. The curve of the beam S1_C45_A60_ϕ5_1 shows a brittle response, because *M*_u_ < *M*_cr_*, whereas the reinforcement of S1_C45_A60_ϕ5_2 is close to the minimum value as *M*_u_ ≅ *M*_cr_*. Finally, the *M*-$\bar{w}$ curve of S1_C45_A60_ϕ5_3 describes a typical ductile behavior with *M*_u_ > *M*_cr_*. The same behavior can also be observed in the beams of Group 10, which are reported in Figure 7c,d.

### Numerical Brittle/Ductile Assessment

As in the case of LRC and FRC beams [15,21,37], the brittle/ductile behavior of HRC beams can be evaluated by means of the following ductility index (*DI*)

(25) $$DI=\frac{M_{u}-M_{\mathrm{cr}*}}{M_{\mathrm{cr}*}}=\frac{P_{u}-P_{\mathrm{cr}*}}{P_{\mathrm{cr}*}}$$

Based on Equation (1), *DI* assumes positive values for lightly reinforced beams, showing a ductile response (i.e., when the failure of reinforcement does not occur), whereas under-reinforced concrete members exhibit *DI* < 0. Accordingly, the minimum amount of hybrid reinforcement (or, equivalently, the brittle/ductile transition) can be identified by imposing *DI* = 0.

Since both *M*_u_ (or *P*_u_) and *M*_cr_* (or *P*_cr_*) depend on the amount of reinforcement in HRC beams, *DI* should be in turn a function of *A*_s_ and *V*_f_. In this regard, the following reinforcement ratio *r* can be introduced as the parameter governing the brittle/ductile transition [41]

(26) $$r=\frac{A_{s}}{A_{s,\min}}+\frac{V_{f}}{V_{f,\min}}$$

Specifically, the area *A*_s_ and the fiber content *V*_f_ of HRC beam are linearly combined with the minimum amounts *A*_s,min_ and *V*_f,min,_ coming from the corresponding LRC and FRC beams. In other words, *A*_s,min_ is the area of rebar defined by the brittle/ductile transition of the concrete beam, when it is reinforced with only steel rebar. Analogously, *V*_f,min_ is the fiber volume fraction necessary to satisfy the requirement of Equation (1) in fiber-reinforced concrete beams. As *A*_s,min_ and *V*_f,min_ are defined for the specific type of beam, all the related parameters are automatically taken into account within *r*. In such a way, the non-dimensional variable *r* is normalized with respect to any geometrical and mechanical property. In particular, *r* = 1 for LRC beam reinforced with *A*_s_ = *A*_s,min_ [15], as well as for a FRC beam containing a quantity of fibers *V*_f_ = *V*_f,min_ [21]. Thus, in concrete beams, under-bending actions, *A*_s,min_ and *V*_f,min_ assume the same mechanical function, according to Fantilli et al. [37].

The definition of *r* given by Equation (26) is also in agreement with the findings of Falkner and Henke [33], who demonstrated that the effects produced by rebar and fibers in HRC members can be superposed at ultimate limit state. Hence, *M*_u_ (or *P*_u_), and *DI* as well, can be considered a function of *r*, if *M*_cr_* (or *P*_cr_*) does not vary with the amount of reinforcement.

As in the case of LRC and FRC beams, within each group of beams (e.g., those of Group 9 in Figure 7), a linear relationship between *DI* and *r* is attained (see Figure 7b) and the intersection between the line *DI*-*r* and the horizontal axis (i.e., *DI* = 0) occurs when *r* ≅ 1, corresponding to the minimum hybrid reinforcement. Thus, the following symbolic equation can be written

(27) DI=ζ⋅(r−1)

where the slope ζ is equal to 1 in the presence of only rebar [15], and ζ = 0.7 in FRC beams [21].

**Figure 8:**
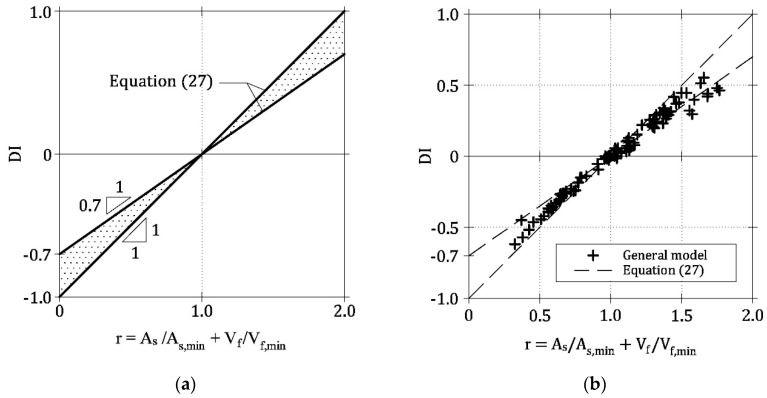
(**a**) Proposed *DI*-*r* envelope for HRC beams [Equation (27)] and (**b**) comparison with the results of the general model.

If LRC and FRC beams are considered as two limit cases, a range delimited by two lines of Equation (27) (i.e., with ζ = 1 and ζ = 0.7) defines the *DI*-*r* relationships of HRC beam (Figure 8a). Indeed, by reporting in a single diagram all the [*DI*, *r*] couples computed in the ideal HRC beams of Table 1, almost all of them fall within this range (Figure 8b). The slope ζ of the least square regression line of all the numerical data is equal to 0.8, and, therefore, it is comprised between the values computed for LRC and FRC beams [15,21].

## 4. Experimental Investigation

To check the effectiveness of the range depicted in Figure 8, an experimental campaign was carried out, in cooperation with Cemex Research Group, with the aim of studying the flexural behavior of HRC beams. Several combinations of rebar and fibers were adopted to reinforce 30 concrete beams. As for conventional reinforcement, steel rebar having ϕ_s_ = 6 mm, *f*_y_ = 527 MPa, and *f*_u_ = 623 MPa were used. Moreover, two types of steel fibers with hooked-ends were used in six different concrete mixtures: short fibers-Type 1 (ϕ_f_ = 0.38 mm, *L*_f_ = 30 mm, and *f*_u_ = 3070 MPa), and long fibers-Type 2 (ϕ_f_ = 0.71 mm, *L*_f_ = 60 mm, and *f*_u_ = 2600 MPa). The compositions of the mixtures (labelled with the letters from A to F) are reported in Table 2. In particular, for both the types of fiber, a reference plain concrete and two FRC mixtures (with *V*_f_ = 0.50 and 0.75%) were tailored by mixing the components in 100 L planetary mixer for 180 s.

**Figure 9:**
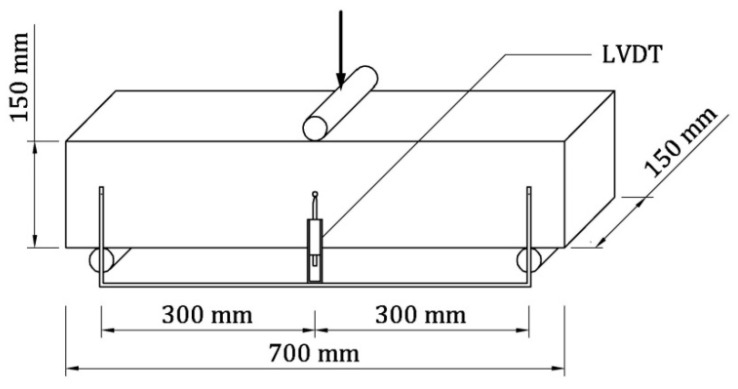
Three-point bending test on un-notched prismatic specimen carried out in the experimental investigation on HRC beams.

As reported in Table 3, LRC beams were cast for the two mixtures without fibers (i.e., series A and series D), whereas both FRC and HRC beams were made with all the other mixtures (i.e., series B, C, E, and F). With these mixtures, 10 series of three un-notched prismatic beams, having a length of 700 mm and a square cross-section of 150 × 150 mm, were cast (Figure 9). Such beams are equal to those tested by Falkner and Henke [33] in four-point bending. Each series of beams was labelled by two letters, referred to the concrete mixture and to the presence (R), or the absence (P), of a single rebar (*A*_s_ = 28 mm^2^).

The beams were tested in three-point bending by using an MTS testing machine. As linear supports (at a distance of 600 mm), and for the application of load as well, steel cylinders were used (see Figure 9). A load cell of 100 kN was used to apply the load *P*, and two LVDTs measured the midspan deflection δ on the two sides of the beam (depurated by the support settlements). The bending tests were performed under displacement control, at a velocity of 0.08 mm per minute up to the maximum load. Afterword, the velocity increased to 0.20 mm per minute.

**Figure 10:**
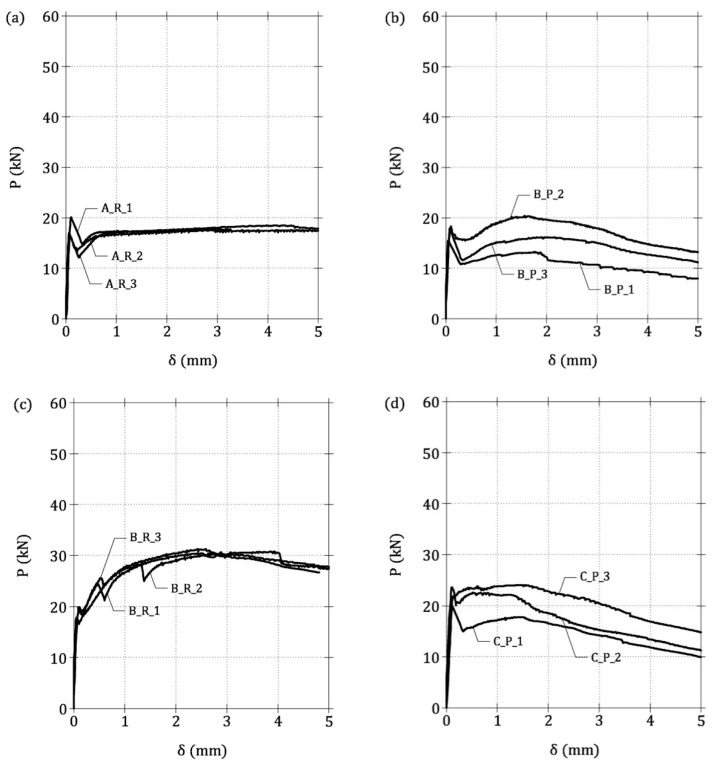

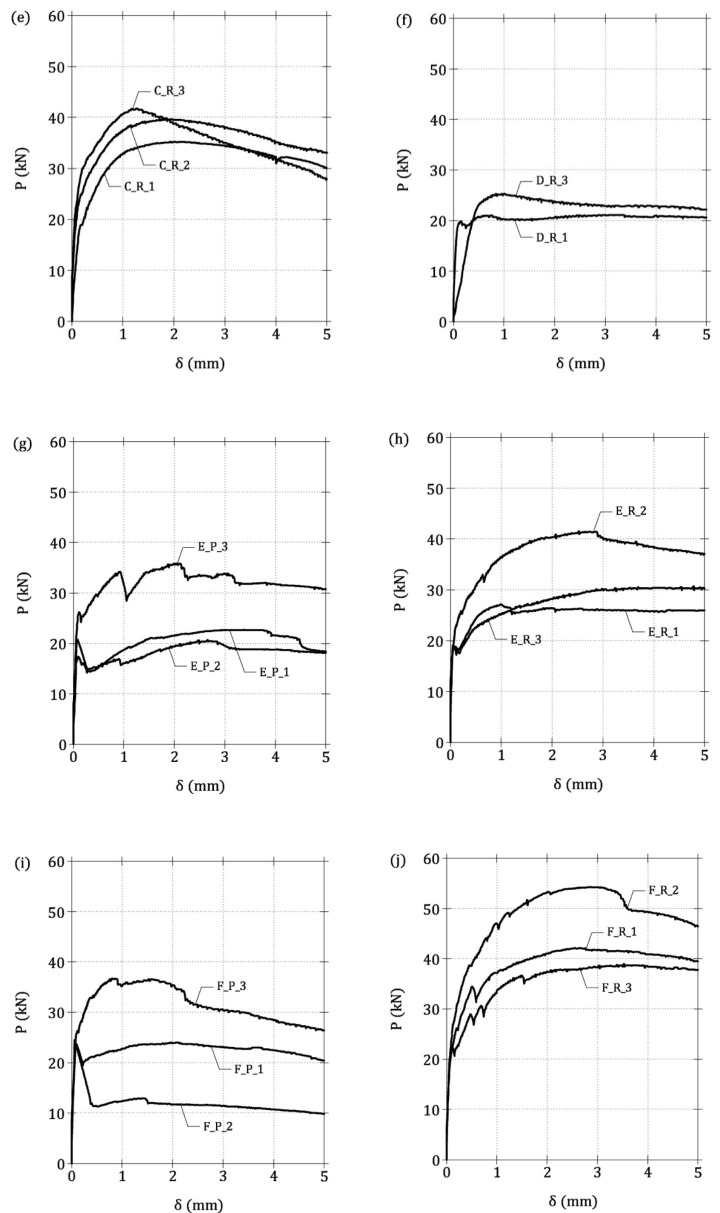
Load vs. deflection curves from three point bending tests. (**a**) A_R series, (**b**) B_P series, (**c**) B_R series, (**d**) C_P series, (**e**) C_R series, (**f**) D_R series, (**g**) E_P series, (**h**) E_R series, (**i**) F_P series and (**j**) F_R.

To measure the compressive strength of the mixtures, cylindrical specimens (with a diameter of 150 mm and a height of 300 mm) were also tested in uniaxial compression. The cylinders were tested 28 days after casting with a Galdabini testing machine, having a load capacity of 5000 kN. During all the test, the velocity of the stroke was kept constant, at 0.60 mm per minute. The *P*-δ curves of the 30 concrete beams are illustrated in Figure 10, where they are grouped in the 10 series of beams as described in Table 3.

Specifically, Figure 10a represents the curves of the LRC beams cast with mixture A, whereas the curves of the beams containing short fibers (i.e., mixtures B and C), with and without rebar, are shown in Figure 10b–e. Similarly, Figure 10f illustrates the *P*-δ curves of the LRC beams made with the mixture D, and in Figure 10g–j the mechanical responses of the beams containing long fibers (i.e., mixtures E and F), with and without rebar, are shown.

Concerning the LRC beams (i.e., A_R and D_R), a ductile behavior can be observed in the diagrams of Figure 10a,f, because the beams were able to bear the maximum load after cracking. For the FRC beams with short and long fibers (i.e., B_P, C_P, E_P and F_P), the softening branch after the cracking is followed by an hardening stage, as depicted in the *P*-δ curves of Figure 10b,d,g,i, respectively. On the other hand, when a rebar is added to the previous elements, the HRC beams (i.e., B_R, C_R, E_R and F_R) exhibit a clear deflection hardening (post-cracking load is greater than cracking load in Figure 10c,e,h,j). A certain dispersion of the experimental data can be noticed in the diagrams of Figure 10, especially in the beams B_P, C_P, E_P, and F_P. In these beams, due to the absence of rebar, the random dispersion and orientation of fibers play a fundamental role on the post-cracking bearing capacity. Such a dispersion is larger in beams having a small width and depth (only 150 mm in this case).

### Experimental Brittle/Ductile Assessment

According to Fantilli et al. [37], the minimum amount of reinforcement of both LRC and FRC beams can be determined by applying the same design-by-testing approach, summarized by the following formulae

(28) $$A_{s,\min}=\frac{\zeta \cdot A_{s}}{DI+\zeta }$$

(29) $$V_{f,\min}=\frac{\zeta \cdot V_{f}}{DI+\zeta }$$

where *A*_s_ and *V*_f_ are the amounts of rebar and fibers in the tested beam, and ζ can be assumed, for the sake of the simplicity, 0.8 for both LRC and FRC beams. Hence, the values of *A*_s,min_ and *V*_f,min_ can be determined for the LRC and FRC beams associated to the HRC beam, making, in turn, the evaluation of *r* (with Equation (26)) be possible.

In addition to the specimens tested herein, this procedure is also applied to the results of some experimental campaigns on HRC elements in bending performed by Carpinteri et al. [42], Dancygier and Berkover [30], di Prisco et al. [31], Dupont [32], Falkner and Henke [33], and You et al. [34]. With the exception of the three-point bending tests carried out by Carpinteri et al. [42], in all the other experimental investigations, the beams were tested in four-point bending. For each beam, *DI* is calculated with Equation (25) and, after computing *A*_s,min_ and *V*_f,min_ for HRC beams (Equations (28) and (29)), *r* is also evaluated (Equation (26)). The values of *DI* and *r* are reported in Table 4.

**Figure 11:**
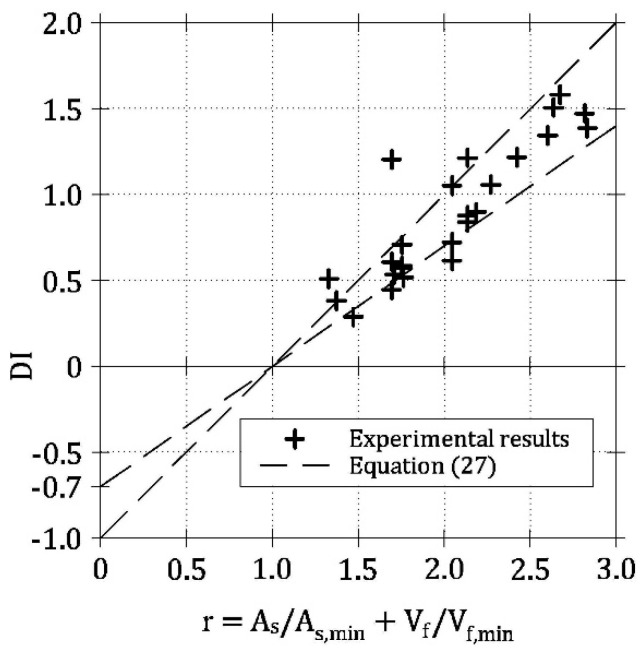
Proposed *DI*-*r* range [Equation (27)] and the results of some experimental campaigns.

The experimental values of *DI*, obtained for 25 HRC beams, are plotted in Figure 11 as a function of *r*. In this figure, experimental data are compared to the range defined by Equation (27) when ζ = 0.7 and ζ = 1. According to the numerical results, as most of the points representing the experimental data fall within the range, the brittle/ductile transition (i.e., *DI* = 0) really occurs when *r* ≅ 1. Therefore, the simplified hypotheses used in the general model (fiber symmetrically and orthogonally positioned with respect to crack surfaces, linear crack profile in HRC beam, etc.) seem to be irrelevant to assess the brittle/ductile behavior of HRC beams, as already found by Fantilli et al. [15,21] in LRC and FRC beams.

**Figure 12:**
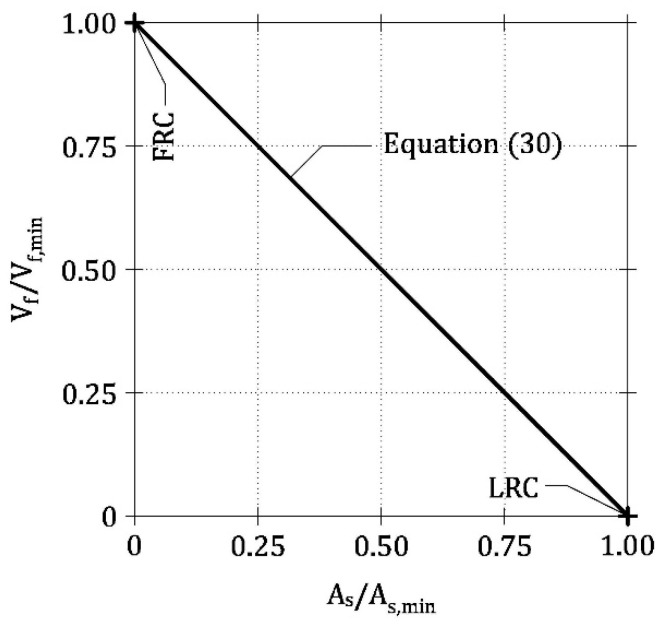
The condition of minimum ductility (i.e., DI = 0) given by Equation (30), when rebar and fibers are used to reinforce concrete beams.

From a practical point of view, it is useful to analyze all the ways to reinforce HRC beam in order to satisfy the requirement *P*_u_ = *P*_cr_* or *DI* = 0. Indeed, it is sufficient to impose *r* = 1 into Equation (26), as revealed by both numerical and experimental results (Figure 8b and Figure 11, respectively). Accordingly, the minimum reinforcement to be used in HRC members is given by any linear combination of *A*_s,min_ and *V*_f,min_ (Figure 12)

(30) $$\frac{A_{s}}{A_{s,\min}}+\frac{V_{f}}{V_{f,\min}}=1$$

Hence, by combining rebar and fibers, it is possible to reduce the minimum amount of reinforcement *A*_s,min_ traditionally required by building codes for LRC beams [18,19,20]. This is in accordance with the results of some previous theoretical models [35,36] and with the recommendations given by Model Code 2010 [20]. In addition, Equation (30) is similar to the formulation proposed by Liao et al. [3], even if the terms *A*_s,min_ and *V*_f,min_ are herein evaluated by testing LRC and FRC full-scale members, rather than small beam specimens. On the other hand, the use of non-dimensional parameters in Equations (25)–(30) makes the geometrical dimensions of the beam irrelevant. This is a novelty of the proposed approach, which correctly predicts the experimental results without including any size-effect law [42,43]. As a consequence, in design-by-testing approaches, used to evaluate the minimum hybrid reinforcement of real structures [44], the transition between small specimens and real structures is automatically included within the ductility index.

## 5. Conclusions

The numerical and experimental analyses reported in this paper lead to the following conclusions:

1. The brittle/ductile flexural behavior of hybrid reinforced concrete beams HRC can be described by the ductility index *DI*, which, in turn, depends on the difference between the ultimate load and the effective cracking load of a beam;
2. The hybrid reinforcement of lightly reinforced concrete beams can be quantified by means of *r*, which is a linear combination of the area of rebar and the volume of fibers_,_ both normalized with respect to the minimum reinforcement of LRC and FRC beams, respectively;
3. Both numerical and experimental investigations performed on HRC beams reveal the existence of a range in the *DI* vs. *r* diagram. The borders of this range are two linear *DI*-*r* functions reproducing the behavior of LRC and FRC beams, respectively;
4. The minimum hybrid reinforcement, corresponding to *DI* = 0, is a linear combination of the minimum amount of rebar and the minimum fiber volume fraction required for LRC and FRC beams, separately. Accordingly, the minimum reinforcement of LRC beams can be reduced by the presence of fibers.

Further theoretical and experimental studies will be devoted to extending the current approach to HRC beams under shear and bending actions, and to the brittle/ductile response of other structures (e.g., slabs on ground).

## Figures and Tables

**Table 1:** Properties of the ideal HRC beams of Groups 1–36.

Group	Beam	*H* (mm)	*f*_c_ (MPa)	*L*_f_/ϕ_f_	ϕ_s_ (mm)	*A*_s_ (mm^2^)	*V*_f_ (%)
1	S1_C30_A80_ϕ4_1	200	30	80	4	13	0.15
	S1_C30_A80_ϕ4_2					25	0.15
	S1_C30_A80_ϕ4_3					38	0.15
2	S1_C30_A80_ϕ5_1				5	20	0.05
	S1_C30_A80_ϕ5_2					20	0.20
	S1_C30_A80_ϕ5_3					20	0.35
3	S1_C30_A60_ϕ4_1			60	4	13	0.25
	S1_C30_A60_ϕ4_2					25	0.25
	S1_C30_A60_ϕ4_3					38	0.25
4	S1_C30_A60_ϕ5_1				5	20	0.15
	S1_C30_A60_ϕ5_2					20	0.30
	S1_C30_A60_ϕ5_3					20	0.45
5	S1_C30_A40_ϕ4_1			40	4	13	0.30
	S1_C30_A40_ϕ4_2					25	0.30
	S1_C30_A40_ϕ4_3					38	0.30
6	S1_C30_A40_ϕ5_1	200	30		5	20	0.10
	S1_C30_A40_ϕ5_2					20	0.40
	S1_C30_A40_ϕ5_3					20	0.70
7	S1_C45_A80_ϕ5_1		45	80	5	20	0.15
	S1_C45_A80_ϕ5_2					39	0.15
	S1_C45_A80_ϕ5_3					59	0.15
8	S1_C45_A80_ϕ6_1				6	28	0.10
	S1_C45_A80_ϕ6_2					28	0.25
	S1_C45_A80_ϕ6_3					28	0.40
9	S1_C45_A60_ϕ5_1			60	5	20	0.10
	S1_C45_A60_ϕ5_2					39	0.10
	S1_C45_A60_ϕ5_3					59	0.10
10	S1_C45_A60_ϕ6_1				6	28	0.05
	S1_C45_A60_ϕ6_2					28	0.25
	S1_C45_A60_ϕ6_3					28	0.45
11	S1_C45_A40_ϕ5_1			40	5	20	0.15
	S1_C45_A40_ϕ5_2					39	0.15
	S1_C45_A40_ϕ5_3					59	0.15
12	S1_C45_A40_ϕ6_1				6	28	0.10
	S1_C45_A40_ϕ6_2					28	0.40
	S1_C45_A40_ϕ6_3					28	0.70
13	S1_C60_A80_ϕ5_1		60	80	5	20	0.15
	S1_C60_A80_ϕ5_2					39	0.15
	S1_C60_A80_ϕ5_3					59	0.15
14	S1_C60_A80_ϕ6_1				6	28	0.10
	S1_C60_A80_ϕ6_2					28	0.25
	S1_C60_A80_ϕ6_3					28	0.40
15	S1_C60_A60_ϕ5_1			60	5	20	0.25
	S1_C60_A60_ϕ5_2					39	0.25
	S1_C60_A60_ϕ5_3					59	0.25
16	S1_C60_A60_ϕ6_1				6	28	0.10
	S1_C60_A60_ϕ6_2					28	0.35
	S1_C60_A60_ϕ6_3					28	0.60
17	S1_C60_A40_ϕ5_1			40	5	20	0.30
	S1_C60_A40_ϕ5_2					39	0.30
	S1_C60_A40_ϕ5_3					59	0.30
18	S1_C60_A40_ϕ6_1				6	28	0.10
	S1_C60_A40_ϕ6_2					28	0.50
	S1_C60_A40_ϕ6_3					28	0.90
19	S2_C30_A80_ϕ8_1	400	30	80	8	50	0.10
	S2_C30_A80_ϕ8_2					101	0.10
	S2_C30_A80_ϕ8_3					151	0.10
20	S2_C30_A80_ϕ10_1				10	79	0.05
	S2_C30_A80_ϕ10_2					79	0.20
	S2_C30_A80_ϕ10_3					79	0.35
21	S2_C30_A60_ϕ8_1	400	30	60	8	50	0.15
	S2_C30_A60_ϕ8_2					101	0.15
	S2_C30_A60_ϕ8_3					151	0.15
22	S2_C30_A60_ϕ10_1	400	30		10	79	0.05
	S2_C30_A60_ϕ10_2					79	0.20
	S2_C30_A60_ϕ10_3					79	0.35
23	S2_C30_A40_ϕ8_1			40	8	50	0.35
	S2_C30_A40_ϕ8_2					101	0.35
	S2_C30_A40_ϕ8_3					151	0.35
24	S2_C30_A40_ϕ10_1				10	79	0.10
	S2_C30_A40_ϕ10_2					79	0.50
	S2_C30_A40_ϕ10_3					79	0.90
25	S2_C45_A80_ϕ8_1		45	80	8	50	0.15
	S2_C45_A80_ϕ8_2					151	0.15
	S2_C45_A80_ϕ8_3					251	0.15
26	S2_C45_A80_ϕ10_1				10	79	0.10
	S2_C45_A80_ϕ10_2					79	0.35
	S2_C45_A80_ϕ10_3					79	0.60
27	S2_C45_A60_ϕ8_1			60	8	50	0.10
	S2_C45_A60_ϕ8_2					151	0.10
	S2_C45_A60_ϕ8_3					251	0.10
28	S2_C45_A60_ϕ10_1				10	79	0.10
	S2_C45_A60_ϕ10_2					79	0.45
	S2_C45_A60_ϕ10_3					79	0.80
29	S2_C45_A40_ϕ8_1			40	8	50	0.10
	S2_C45_A40_ϕ8_2					151	0.10
	S2_C45_A40_ϕ8_3					251	0.10
30	S2_C45_A40_ϕ10_1				10	79	0.10
	S2_C45_A40_ϕ10_2					79	0.60
	S2_C45_A40_ϕ10_3					79	1.10
31	S2_C60_A80_ϕ8_1		60	80	8	50	0.25
	S2_C60_A80_ϕ8_2					151	0.25
	S2_C60_A80_ϕ8_3					251	0.25
32	S2_C60_A80_ϕ10_1				10	79	0.10
	S2_C60_A80_ϕ10_2					79	0.35
	S2_C60_A80_ϕ10_3					79	0.60
33	S2_C60_A60_ϕ8_1			60	8	50	0.10
	S2_C60_A60_ϕ8_2					151	0.10
	S2_C60_A60_ϕ8_3					251	0.10
34	S2_C60_A60_ϕ10_1				10	79	0.10
	S2_C60_A60_ϕ10_2					79	0.50
	S2_C60_A60_ϕ10_3					79	0.90
35	S2_C60_A40_ϕ8_1			40	8	50	0.10
	S2_C60_A40_ϕ8_2					151	0.10
	S2_C60_A40_ϕ8_3					251	0.10
36	S2_C60_A40_ϕ10_1				10	79	0.10
	S2_C60_A40_ϕ10_2					79	0.75
	S2_C60_A40_ϕ10_3					79	1.40

**Table 2:** Material components (in 1 m^3^) of the concrete mixtures used in this research project.

Mixture	CEM I 52.5R (kg)	Water (l)	Sand 0–4 mm (kg)	Gravel 4–8 mm (kg)	Gravel 8–11 mm (kg)	Super-plasticizer (kg)	*V*_f_ Type 1 (%)	*V*_f_ Type 2 (%)
A	400	200	864	346	519	3.2	0.00	0.00
B						4.0	0.50	0.00
C							0.75	0.00
D						3.2	0.00	0.00
E						4.0	0.00	0.50
F							0.00	0.75

**Table 3:** Amounts of rebar and fibers used to reinforce the beams tested in this research project.

Mixture	Beam	*A*_s_ (mm^2^)	*V*_f_ (%)
A	A_R	28	0.00
B	B_P	0	0.50
	B_R	28	
C	C_P	0	0.75
	C_R	28	
D	D_R	28	0.00
E	E_P	0	0.50
	E_R	28	
F	F_P	0	0.75
	F_R	28	

**Table 4:** Evaluation of *r* and *DI* in the HRC beams tested in this research project and in other experimental campaigns.

Beam	*A*_s,min_ (mm^2^)	*V*_f,min_ (%)	*r*	*P*_cr_* (kN)	*P*_u_ (kN)	*DI*	Ref.
A_R_1	29	0.66	0.99	20.09	17.70	−0.12	Tested herein
A_R_2				16.52	17.71	0.07	
A_R_3				17.02	18.51	0.09	
B_P_1			0.76	15.44	13.27	−0.14	
B_P_2				18.26	20.39	0.12	
B_P_3				17.84	16.17	−0.09	
B_R_1			1.75	17.84	30.44	0.71	
B_R_2				19.44	30.79	0.58	
B_R_3				19.92	31.31	0.57	
C_P_1			1.14	20.06	17.82	−0.11	
C_P_2				21.90	22.58	0.03	
C_P_3				23.67	24.10	0.02	
C_R_1			2.13	18.78	35.24	0.88	
C_R_2				17.91	39.65	1.21	
C_R_3				22.72	41.76	0.84	
D_R_1		0.71	0.99	19.84	21.14	0.07	
D_R_2				MISSING			
D_R_3				25.26	22.99	−0.09	
E_P_1			0.70	17.37	22.70	0.31	
E_P_2				20.79	20.72	0.00	
E_P_3				26.17	35.83	0.37	
E_R_1			1.69	18.82	27.17	0.44	
E_R_2				18.86	41.55	1.20	
E_R_3				19.08	30.69	0.61	
F_P_1			1.05	23.69	24.01	0.01	
F_P_2				24.47	12.93	−0.47	
F_P_3				26.31	36.70	0.39	
F_R_1			2.04	26.11	42.13	0.61	
F_R_2				26.45	54.27	1.05	
F_R_3				22.63	38.94	0.72	
0−1ϕ8	41	0.53	1.23	16.31	19.33	0.19	[42]
40−0ϕ0			0.96	16.05	15.47	−0.04	
10−1ϕ8			1.47	16.70	21.48	0.29	
20−1ϕ8			1.71	16.64	25.52	0.53	
40−1ϕ8			2.19	14.35	27.22	0.90	
NF−0−015	40	6.66	2.52	17.25	38.24	1.22	[30]
NF−1−015			2.63		43.24	1.51	
Slag	106	3.88	0.31	31.12	13.93	−0.55	[31]
Slag R/C			1.37	35.39	48.88	0.38	
Filler	101	5.80	0.21	32.64	11.93	−0.63	
Filler R/C			1.33	31.07	46.88	0.51	
1 + 2	52	0.43	0.74	27.22	21.62	−0.21	[32]
13 + 14			2.67	26.55	68.50	1.58	
3 + 4	54	0.65	0.98	27.56	27.22	−0.01	
25			2.83	32.51	77.60	1.39	
7 + 8	47	0.71	0.45	10.32	5.77	−0.44	
20			2.60	11.33	26.56	1.34	
9 + 10	92	0.54	1.17	14.84	16.90	0.14	
27			2.27	16.40	33.74	1.06	
RC	24	0.88	1.18	3.77	4.32	0.15	[33]
SFRC			0.58		2.51	−0.33	
RC/SFRC			1.76		5.72	0.52	
A	48	0.00	2.08	54.09	100.90	0.87	[34]
ASF50LD80		1.88	2.42	57.40	127.17	1.22	
ASF40LD65TF4		1.12	2.81	58.76	145.27	1.47	
